# Systems Biology-Based Identification of Crosstalk between E2F Transcription Factors and the Fanconi Anemia Pathway

**Published:** 2007-05-01

**Authors:** Moe Tategu, Takako Arauchi, Rena Tanaka, Hiroki Nakagawa, Kenichi Yoshida

**Affiliations:** Department of Life Sciences, Meiji University School of Agriculture, 1-1-1 Higashimita, Tama-ku, Kawasaki, Kanagawa 214-8571, Japan

**Keywords:** E2F, Fanconi anemia, cell cycle, DNA damage/repair, transcriptional regulation, bioinformatics

## Abstract

Fanconi anemia (FA) is an autosomal recessive disorder characterized by congenital abnormalities, bone marrow failure, chromosome fragility, and cancer susceptibility. At least eleven members of the *FA* gene family have been identified using complementation experiments. Ubiquitin-proteasome has been shown to be a key regulator of FA proteins and their involvement in the repair of DNA damage. Here, we identified a novel functional link between the FA/BRCA pathway and E2F-mediated cell cycle regulome. *In silico* mining of a transcriptome database and promoter analyses revealed that a significant number of *FA* gene members were regulated by E2F transcription factors, known to be pivotal regulators of cell cycle progression – as previously described for *BRCA1*. Our findings suggest that E2Fs partly determine cell fate through the FA/BRCA pathway.

## Introduction

Fanconi anemia (FA) is an autosomal recessive disorder characterized by congenital abnormalities, bone marrow failure, chromosome fragility, and cancer susceptibility (Joenje and Patel, 2001). To date, at least eleven members of this family, including *FANCA*, *FANCB*, *FANCC*, *FANCD1* (alias *BRCA2*), *FANCD2*, *FANCE*, *FANCF*, *FANCG* (alias *XRCC9*), *FANCJ*, *FANCL*, and *FANCM* (Joenje and Patel 2001), have been identified using complementation experiments. Among them, eight FA-associated genes have been identified so far (*FANCA*, *FANCC*, *FANCD1*, *FANCD2*, *FANCE*, *FANCF*, *FANCG*, and *FANCL* (Strathdee et al. 1992; Joenje and Patel, 2001; Timmers et al. 2001; Howlett et al. 2002; Meetei et al. 2003). The FANCA, FANCC, FANCE, FANCF, FANCG, and FANCL proteins are part of a nuclear multiprotein core complex that triggers the monoubiquitination of the FANCD2 protein during S phase of the cell cycle and after exposure to DNA crosslinking agents (Garcia-Higuera et al. 2001; Meetei et al. 2003; Soulier et al. 2005). Monoubiquitinated FANCD2 colocalizes in nuclear foci with BRCA1, FANCD1, and NBS1 (Garcia-Higuera et al. 2001; Howlett et al. 2002; Nakanishi et al. 2002). This evidence suggests a link between the FA protein complex and the cellular BRCA1 repair machinery. Therefore, the FA/BRCA pathway is thought to be involved in the repair of DNA damage to maintain genomic integrity (D’Andrea and Grompe, 2003). The cellular targets of the FA/BRCA pathway remain unknown.

A remarkably high clinical variability exists among FA patients (Auerbach et al. 2001; Alter, 2003). Besides the ubiquitination of FA proteins, other regulatory mechanisms may affect the expression level of FA proteins; such regulatory mechanisms may contribute to the clinical variability observed among FA patients. Bioinformatics is a useful tool for integrating complex gene functions. It also allows the establishment of biological frameworks or “system biology”. It is believed to connect between gene regulation and phenotypes. Our current standard concept for gene regulation can be roughly divided into two aspects; namely, gene transcription and protein degradation. If the significant regulators of gene transcription or protein degradation for each component of complex gene networks could be revealed, one could easily imagine the gene network and extract the phenotypes of cells or model organisms. As a logical consequence, system biology views are an unavoidable necessity for current biology studies.

We have focused on the E2F transcription factor-regulated transcriptome because E2F family members integrate the upstream signals to the downstream target genes during the monitoring of proper cell cycle progression. Indeed, the downstream target genes of E2F1 can be divided into several categories including DNA replication, DNA damage/repair, apoptosis, differentiation, and development (Bracken et al. 2004). Evidence that E2F family members regulate *BRCA1* expression and interact with BRCA1 protein (Wang et al. 2000; Oberley et al. 2003; Bindra and Glazer, 2006) prompted us to investigate comprehensively the relationship between E2Fs and *FA* genes.

In the present study, we evaluated the possible link between E2F transcription factors and *FA* genes by analyzing the *FA* gene promoters *in silico* followed by a luciferase-based promoter assay. These analyses, combined with a public microarray data search, allowed us to identify a novel aspect of the FA pathway that is partially regulated in a cell cycle-dependent manner via E2Fs. The discovery that both *FA* genes and *BRCA1* are under the control of E2Fs suggests that the FA/BRCA pathway is an effector of E2F-regulated cell cycle progression and DNA damage/repair signaling. Comprehensive regulome analyses of the *FA* gene using cell cycle-associated transcriptional factors may enable us to open a new window onto the system biology of *FA* genes.

## Materials & Methods

### Bioinformatics

The E2F1 gene expression profile identified using adenovirus-mediated gene transfer in SKMEL-2 melanoma cells (Affymetrix GeneChip analysis (Jamshidi-Parsian et al. 2005)) was deposited in the Gene Expression Omnibus (GEO), which is maintained by The National Center for Biotechnology Information (NCBI, NIH) (http://www.ncbi.nlm.nih.gov/). By searching the database, *FANCA* (Probe No. 33145), *FANCC* (Probe Nos. 35713, 1982_s, and 160034_s), *FANCD1* (Probe Nos. 1503, 1989, and 1990_g), *FANCG* (Probe No. 37584), and *FANCL* (Probe No. 33125) mRNAs were identified (Accession No. GDS1078; Platform No. GPL91). The expression profile suggested by an analysis of the EST (expressed sequence tag) counts in human tissues and organs was searched using the UniGene database (EST Profile Viewer, NCBI, NIH). The data sets used for the *FA* genes are shown in Table 1. The number of transcripts per million was calculated based on the gene EST/total EST in the pool, and this value was exported to an Excel file. Transfac software (http://motif.genome.jp/) was then used to determine the E2F family of transcription factors binding-elements.

### Plasmids

The human *FA* promoter fragments were generated using polymerase chain reaction (PCR) from genomic DNA, and ligated into pGL3-Basic vectors (Promega, Madison, WI, U.S.A). PCR primers were designed to amplify 318-bp (−274/+44), 333-bp (−263/+70), 298-bp (−232/+66), 318-bp (−286/+32), 224-bp (−208/+16), 1330-bp (−1287/+43), 328-bp (−373/+37), 442-bp (−441/+1), 256-bp (−249/+7), 860-bp (−835/+25), and 410-bp (−732/−128) fragments of the human *FANCA*, *FANCB*, *FANCC*, FANCD1, FANCD2, FANCE, FANCF, FANCG, *FANCJ*, *FANCL*, and *FANCM* promoter sequences, respectively; the above numbering is relative to the transcription initiation site at +1, described in the UniGene Database (Genome View, NCBI, NIH). The GenBank Accession numbers of the genomic clones used for the designation of the PCR primers are shown in Table 1. A KpnI site was added to the forward primer and a BglII site was added to the reverse primer to facilitate the subcloning of the promoter regions of *FANCA*, *FANCB*, *FANCC*, FANCD1, FANCD2, FANCF, FANCG, FANCJ, *FANCL*, and *FANCM*, respectively. A KpnI site was added to the forward primer and a HindIII site was added to the reverse primer to facilitate the subcloning of the promoter region of *FANCE*. The following plasmids that were used have been previously described: pcDNA3-E2F1, E2F2, E2F3, E2F4, E2F5, and E2F6 (Yoshida and Inoue, 2004; Hayashi et al. 2006).

### Cell culture and luciferase assay

HeLa and WI-38 cells were cultured in Earle’s modified Eagle’s medium (Invitrogen, Carlsbad, CA, U.S.A) supplemented with 10% fetal bovine serum and antibiotics. Preparation of the adenovirus, the virus infection procedure, and the Western blot analysis were as described previously (Goto et al. 2006). For the promoter assay, 2 × 10^4^ HeLa cells were transfected with FuGENE6, in accordance with the manufacturer’s instructions (Roche Diagnostic, Basel, Switzerland). Briefly, 400 ng of the expression plasmid, 200 ng of the firefly luciferase reporter plasmid (pGL3, Promega, Madison, WI, U.S.A.), and 0.6 ng of the *Renilla* luciferase reporter plasmid (pRL-TK, Promega, Madison, WI, U.S.A.) per 24-well dish were used for each transfection. The cells were harvested 24 hours after the transfection, and a luciferase assay was performed using the Dual-Luciferase Reporter Assay System, in accordance with the manufacturer’s protocol (Promega, Madison, WI, U.S.A.). As a control for transfection efficiency, the firefly luciferase activity values were normalized to the *Renilla* luciferase activity values. Data are presented as the mean values ± standard deviation.

### RT (reverse transcriptase)-PCR

Total cellular RNA was extracted from the WI-38 cells using an RNeasy Mini Kit (Qiagen, Valencia, CA, U.S.A.), in accordance with the manufacturer’s instructions. The RT step was also performed according to the manufacturer’s directions (Invitrogen, Carlsbad, CA, U.S.A.). Briefly, 500 ng of extracted RNA, oligo(dT) primer, and 1 × annealing buffer were diluted in 8 μL of RNase/DNase-free water, heated to 65 °C for 5 minutes, and then chilled on ice. For the first-strand cDNA synthesis, a heat-denatured RNA solution, along with 2 × first-strand reaction mix and the SuperScript III/RNase OUT enzyme mix, was added to make up 20 μL of the reaction mixture, followed by incubation at 50 °C for 50 minutes, and then heating at 85 °C for 5 minutes and cooling on ice. The following primers were designed to amplify 300-bp long *FANCC* (NM_000136) cDNA: sense, 5′-gggcctctctcctgttctga-3′ and antisense, 5′-gaggtcagggcttccaggct-3′. PCR was then performed as follows: denaturation for 2 minutes at 94 °C, followed by 25–30 cycles at 94 °C for 15 seconds, 55 °C for 30 seconds, and 68 °C for 30 seconds. As a control, a *GAPDH* primer set was used (R&D Systems, Minneapolis, MN, U.S.A).

## Results

### Expression profiles of human FA mRNAs

**Figure 1:**
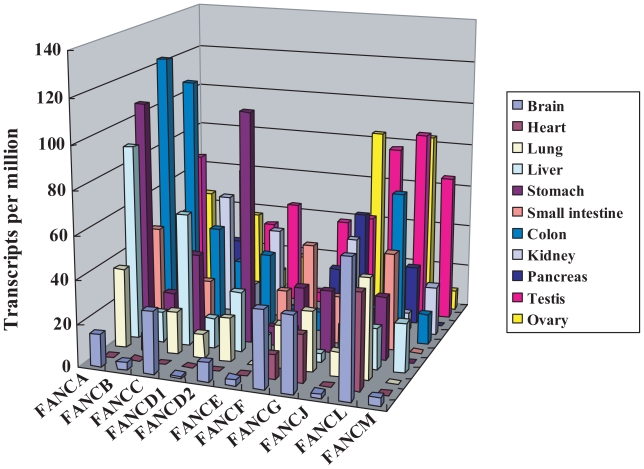
Expression profiles of *FA*. A search was conducted based on the EST counts in human tissues and organs in the UniGene database (NCBI, NIH). The data sets used for the *FA genes* are listed in Table 1. The number of transcripts per million was calculated from the gene EST/total EST in the pool.

To investigate the common expression patterns of the *FA* mRNA, we initially compared the abundance of ESTs among major human tissues and organs including the brain, heart, lung, liver, stomach, small intestine, colon, kidney, pancreas, testis, and ovary. *In silico* analysis revealed that the expression patterns of the *FA* mRNA differed considerably in human tissues and organs (Fig. 1). One characteristic feature of the expression patterns of *FA* genes was that *FA* genes were less expressed in the heart but were relatively well expressed in the stomach, colon, testis, and ovary. We speculated that the expression of FA genes tends to be enriched in proliferative tissues.

**Figure 2:**
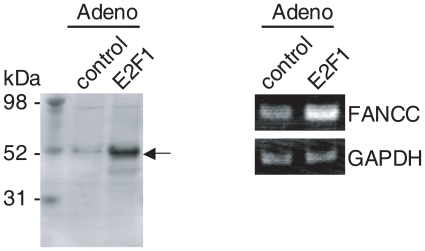
E2F1 activates *FANCC* expression in WI-38 cells. (A) Cultures were infected with adenovirus encoding E2F1 or mock (control), and the E2F1 protein level was determined using a Western blot analysis with an anti-E2F1 antibody. Molecular weights (kDa) are shown on the left. The position of the E2F1 protein is indicated by the arrow on the right. RNA was extracted from WI-38 cells 24 hours after infection and analyzed using RT-PCR for *FANCC* and *GAPDH*.

Next, we asked which transcription factor regulates the FA/BRCA pathway. The E2F family of transcription factors plays pivotal roles in the cell cycle progression and DNA damage repair pathways (Bracken et al. 2004). Therefore, we searched for *FA* mRNAs in public databases where the E2F family of transcription factors was either overexpressed or knocked down. A GEO database search revealed the deposition of microarray data for *FANCA*, *FANCC*, *FANCD1*, *FANCG*, and *FANCL* (see Materials & Methods). *FANCA* and *FANCL* mRNAs were clearly upregulated in E2F1 overexpressed SKMEL-2 melanoma cells, whereas *FANCD1* and *FANCG* mRNAs were unchanged after E2F1 overexpression. On the other hand, the upregulation of *FANCC* mRNA by E2F1 was shown by one probe used in a microarray experiment but not by other probes. To substantiate the E2F1-regulated *FANCC* mRNA expression, human normal lung fibroblast WI-38 cells were infected with an adenovirus expressing E2F1. A Western blot analysis revealed that a faint band of endogenous E2F1 protein was detected in the control adenovirus-infected cell lysates, whereas significant amounts of exogenous E2F1 protein were detected in the E2F1 overexpressed cell lysates (Fig. 2, left panel). Twenty-four hours after the virus infection, the *FANCC* mRNA level was found to be upregulated, while the *GAPDH* mRNA level remained unchanged (Fig. 2, right panel). This result suggests that FANCC could be a downstream target gene of E2F1.

### Transcriptional regulation of human FA genes

**Figure 3:**
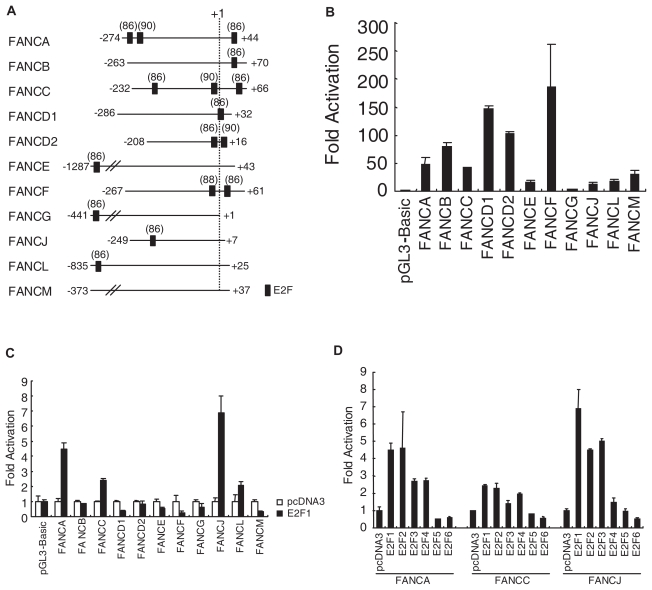
Transcriptional regulation of the human FA genes. (A) Schematic representation of the vicinity of the transcription start sites of the *FA* genes. The putative transcription factor E2F-binding sites (closed box) were searched within 1.5-kbp upstream and 0.5-kbp downstream of the transcription start sites, and are indicated with their scores (maximum score, 100) as calculated by the Transfac program. The transcription start sites (dotted lines) are indicated. The positions of the promoter constructs are numbered relative to the transcription initiation site at +1, as described in the NCBI UniGene database (NCBI, NIH). (B) *FA* promoter activities in asynchronously growing human cells. HeLa cells were transfected with 200 ng of reporter constructs and 400 ng of the expression vector for E2F1, together with 0.6 ng of pRL-TK. The pcDNA3 vector was used as a negative control. At 24 hours after the transfection, the cells were harvested and the extracts were prepared to measure the firefly and *Renilla* luciferase activities. Values are represented as the relative luciferase activities, with that of pGL3-Basic being defined as 1. (C) The E2F-binding motif(s) of the *FANCA, FANCC, FANCJ*, and *FANCL* promoters are sufficient to confer responses to ectopic E2F1 expression. The experiment was performed as described in (B). Values are represented as relative luciferase activities, with that of the control vector pcDNA3 being taken as 1. (D) Activation of the *FA* promoters by members of the E2F family. The experiment was performed as described in (B). Values are represented as the relative luciferase activities, with that of the control vector pcDNA3 being defined as 1.

To gain more insight into the E2F-regulation of *FA* genes, we searched the proximal region of the transcription start site for potential *cis*-elements using Transfac software (ver. 4.0, cut off 85). We focused on the identification of E2F-binding consensus binding sequences within 1.5-kbp upstream and 0.5-kbp downstream of the transcription start site. At least one or up to three E2F-binding consensus sequences were identified for all *FA* genes except the *FANCM* gene (Fig. 3A).

To demonstrate the importance of the putative E2F-binding element for basal promoter activity, we generated promoter-luciferase constructs. These promoter constructs were used to study transient gene expression by transfecting them into HeLa cells and evaluating the firefly luciferase activities by measuring the chemiluminescence with a luminometer. The transfection efficiency was normalized by the dual luciferase assay, in which the corresponding *Renilla* luciferase activity upon co-transfection of the pRL-TK plasmid was also measured. As shown in Figure 3B, the *FA* promoter constructs used in this study showed various extents of increased activity, as determined by measuring the relative luciferase activities, when the activity of the control luciferase vector, pGL3-Basic, was defined as 1. The exogenous coexpression of E2F1 caused up to approximately 4.5-, 2.5-, and 7.0-fold increases in the *FANCA*, *FANCC*, and *FANCJ* promoter activities, respectively, compared to that of the pcDNA3 control vector (Fig. 3C). The promoter regions cloned for *FANCL* were slightly upregulated by the co-expression of E2F1. These results suggest that the E2F-binding motif(s) of the promoter constructs plays critical roles in the E2F1-mediated human *FANCA*, *FANCC*, and *FANCJ* promoter activities.

Next, we sought evidence to show that members of the E2F family transcriptionally regulated the *FANCA*, *FANCC*, and *FANCJ* genes. As shown in Figure 3D, the exogenous co-expression of E2F1 ~ E2F4 caused an increase in the human *FANCA*, *FANCC*, and *FANCJ* promoter constructs, whereas the co-expression of E2F5 or E2F6 was associated with no increase in promoter activity, compared to that in the pcDNA3 control vector. In contrast, the co-expression of E2F1 ~ E2F6 was associated with no increase in pGL3-Basic promoter activity (data not shown).

## Discussion

**Figure 4:**
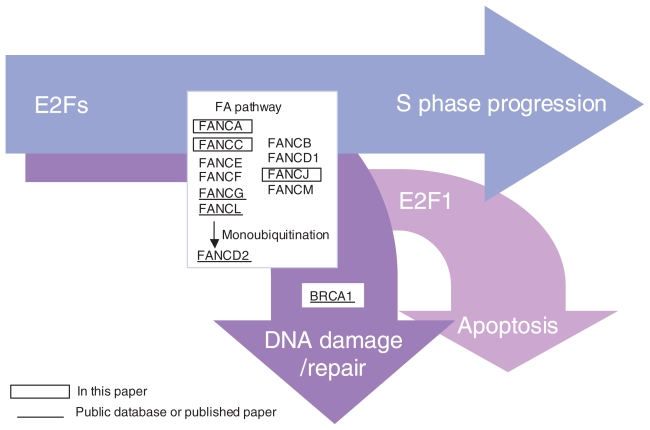
Model depicting the E2Fs-FA/BRCA axis in the control of cell fate. The model shown depicts the E2F-regulated expression of the FA/BRCA pathway as key determinants for cells entering into the DNA damage/repair pathway or the S phase of the cell cycle. Otherwise, cells go into apoptosis, which is exclusively regulated by E2F1 among the E2Fs. The promoter regions cloned for *FANCA, FANCC*, and *FANCJ* were identified as target sequences of E2Fs in the present study (surrounded by box). The regulation of *FANCD2, FANCG*, and *FANCL* as well as *BRCA1* by E2Fs has also been previously reported or deposited in the public database (underlined). As posttranslational events, FANCA, FANCC, FANCE, FANCF, FANCG, and FANCL proteins trigger monoubiquitination of the FANCD2 protein during the S phase of the cell cycle and after DNA damage. Monoubiquitinated FANCD2 colocalizes in nuclear foci with BRCA1, FANCD1, and NBS1.

Each FA has its own characteristic features, but their functions commonly belong to the same categories, such as DNA damage repair or S phase progression. In the present work, we investigated the common transcriptional regulatory factors that regulate *FA* genes. First of all, we examined the abundance of ESTs of *FA* in various human tissues and organs. This approach provided little information regarding the framework of the regulatory network of *FA* genes. Because we had been investigating the transcriptional network of E2F transcription factors, we noted that recent comprehensive gene expression profiling of the E2F transcriptome had pinpointed some *FA* genes under the E2F pathway (Fig. 4). Notably, a microarray approach revealed that *FANCA* could be regulated by E2Fs like E2F1, E2F2, and E2F3 (Vernell et al. 2003). By applying chromatin immunoprecipitation to isolate E2F4-bound sequences, the promoter regions of *FANCD2*, *FANCG*, and *FANCL* were shown to be potentially regulated by E2F4 (Weinmann et al. 2002; Cam et al. 2004). In addition, databases deposited in NCBI revealed that *FANCA*, *FANCL*, and probably *FANCC* genes were upregulated in E2F1-overexpressed SKMEL-2 melanoma cells. We demonstrated that the overexpression of E2F1 in human diploid primary fibroblast WI-38 cells upregulated *FANCC* mRNA expression. This evidence prompted us to examine the extent to which E2Fs contribute to the transcriptional regulation of *FA* genes.

We prospectively analyzed the promoter regions of the eleven known *FA* genes using an *in silico* determination of the putative E2F1 consensus site and promoter analysis based on a luciferase reporter assay. From these studies, the promoter regions cloned for *FANCA*, *FANCC*, and *FANCJ* were found to be upregulated by the co-expression of E2F1; this evidence enabled us to propose a novel gene regulatory network that couples the E2F1 and FA/BRCA pathways (Fig. 4). *FANCD2*, *FANCG*, and *FANCL* may have E2F-responsive sites other than the region used in this study. Promoter analyses for mutations and the methylation status of *FA* genes have been well characterized; these statuses are known to affect the FA/BRCA pathway (Taniguchi et al. 2003). Among *FA* genes, the promoter of *FANCC* has been cloned and shown to be regulated by p53 (Savoia et al. 1995; Liebetrau et al. 1997). Once functional links between the E2F1 and FA/BRCA pathways have been established, the complete system biology of the FA/BRCA pathway could be analyzed by examining their promoter regulation by cell cycle-associated key transcriptional regulators, including p53.

*BRCA1*, a familial breast and ovarian cancer susceptibility gene, encodes nuclear phosphoproteins that function as tumor suppressors in human breast cancer cells. BRCA1 serves as an important negative regulator of the cell cycle through its interaction with E2F transcriptional factors and phosphorylation by cyclins/cdk (cyclin-dependent kinase) complexes (Wang et al. 1997). Moreover, the regulation of *BRCA1* expression by the pRb (retinoblastoma protein)/E2Fs pathway has been extensively characterized (Wang et al. 2000; Oberley et al. 2003; Bindra and Glazer, 2006). The FA/BRCA pathway may be a pivotal effector regulated by activator-E2F signaling under the specific circumstance of DNA damage. Considering the predisposition to neoplasia, pRb mutations were expected to result in activator E2F overexpression; the subsequent expression of FA proteins might then compromise DNA damage, preventing the cells from progressing into a cancer phenotype.

In conclusion, we found that the FA/BRCA pathway is regulated by activator E2Fs responsible for the execution of the DNA damage/repair pathway. Most importantly, this pathway enables mechanistic links between E2F1 and *FA* genes, illuminating the molecular basis of DNA damage/repair and S phase progression. We propose that the present analysis might be used as a research working model to approach system biology, in combination with *in silico* and functional analyses, for a comprehensive characterization of cellular events in any given organism.

## Figures and Tables

**Table 1:** 

Gene	UniGene	Mapping	Genomic clone	Primer sequences
FANCA	Hs.567267	16q24.3	AC005565	F: 5′-GCTTGGTTGGCCAGGTGGT-3′, R: 5′-ATGGCCTTGGCGCCTACAG-3′
FANCB	Hs.554740	Xp22.2	AC140846	F: 5′-AGGCCCTCAGCCTAGGTCC-3′, R: 5′-CAGCGGCAACATACCGGAG-3′
FANCC	Hs.494529	9q22.3	AL157384	F: 5′-AAGAAGCCAGCGCCCCTTC-3′, R: 5′-TGGAATTTTCCCGCGGTCG-3′
FANCD1	Hs.34012	13q12.3	AL445212	F: 5′-CCACCCAAACATGAGCTGG-3′, R: 5′-CTCTGCCGCCTAGTTTCAG-3′
FANCD2	Hs.208388	3p26	AC007999	F: 5′-TGGGCGAGCTTCTCTTCAC-3′, R: 5′-ACTTTCCCGCCAGGCCCGA-3′
FANCE	Hs.302003	6p22-p21	AL022721	F: 5′-CCCGACATCTCCCTTGAAAT-3′, R: 5′-TTGGGAGACCGGAGAAACCC-3′
FANCF	Hs.632151	11p15	AC103801	F: 5′-AAGCGCGGAGACGTTCATG-3′, R: 5′-GCGATCCAGGTGCTGCAGA-3′
FANCG	Hs.591084	9p13	AC004472	F: 5′-GTTAGTTAGGCTGCTTTAC-3′, R: 5′-TGGGTTCCCGCTTCCACCGA-3′
FANCJ	Hs.532799	17q22-q24	AC060798	F: 5′-TGGATGCCGAAGTTCTCGCC-3′, R: 5′-GAAAGGGCACGAGCCCTTCC-3′
FANCL	Hs.646789	2p16.1	AC007250	F: 5′-GGTCAATAAAGATGGGTAGG-3′, R: 5′-TGGGTCCTGCACATGCGCAG-3′
FANCM	Hs.509229	14q21.3	AL121809	F: 5′-GAATGAGGCACGTTAGACGC-3′, R: 5′-AGCTCAACCGCTACGGTTCC-3′
